# Atrial Fibrillation and Dementia: Epidemiological Insights on an Undervalued Association

**DOI:** 10.3390/medicina58030361

**Published:** 2022-03-01

**Authors:** Andrea Saglietto, Andrea Ballatore, Henri Xhakupi, Gaetano Maria De Ferrari, Matteo Anselmino

**Affiliations:** Division of Cardiology, “AOU Città della Salute e della Scienza di Torino” Hospital, Department of Medical Sciences, University of Turin, 10126 Turin, Italy; andrea.saglietto@live.com (A.S.); andrea.ballatore3@gmail.com (A.B.); henri.xhakupi@edu.unito.it (H.X.); gaetanomaria.deferrari@unito.it (G.M.D.F.)

**Keywords:** atrial fibrillation, dementia, Alzheimer’s disease, epidemiology, global burden of disease injuries and risk factors study

## Abstract

*Background and objectives*: Atrial fibrillation (AF) and dementia are growing causes of morbidity and mortality, representing relevant medical and socioeconomic burdens. In this study, based on data from the Global Burden of Disease Injuries and Risk Factors Study (GBD) 2019, we focused on AF and dementia distribution and investigated the potential correlation between the two epidemiological trends. *Materials and Methods*: Crude and age-standardized incidence, prevalence, mortality rate, and disability-adjusted life years (DALYs) lost, derived from GBD 2019, were reported for AF and dementia. Global features were also stratified by high and low sociodemographic-index (SDI) countries. Granger test analysis was performed to investigate the correlation between AF and dementia incidence time trends. *Results*: From 1990 to 2019 crude worldwide incidence and prevalence showed a dramatic increase for both conditions (from 43.24 to 61.01 and from 528.72 to 771.51 per 100,000 individuals for AF, respectively; from 54.60 to 93.52 and from 369.88 to 667.2 per 100,000 individuals for dementia, respectively). In the same timeframe, crude mortality rate doubled for AF and dementia (from 2.19 to 4.08, and from 10.49 to 20.98 per 100,000 individuals, respectively). Age-standardized estimate showed a substantial stability over the years, highlighting the key role of the progressively aging population. Crude estimates of all of the investigated metrics are greater in high SDI countries for both conditions. This association was still valid for age-standardized metrics, albeit by a reduced magnitude, suggesting the presence of higher risk factor burden in these countries. Finally, according to Granger test, we found a significant association between the historical trends of AF and dementia incidence (*p* = 0.004). *Conclusions*: AF and dementia burden progressively increased in the last three decades. Given the potential association between these two conditions, further clinical data assessing this relationship is needed.

## 1. Introduction

Atrial fibrillation (AF) represents the most frequent sustained cardiac arrhythmia in adults [1], with an estimated prevalence of 59.7 million patients worldwide [2]. Alarming epidemiological predictions foresee a further increase of this condition during the next few decades [3]. At the same time, Alzheimer’s disease and other dementias, characterized by memory impairment with at least one additional cognitive dysfunction, constitute an important burden for worldwide health, with an estimated prevalence of 51.6 million patients [4]. It is predicted that this number will increase by 4.6 million new cases every year, doubling in 20 years [5]. Dementia represents a substantial financial burden, comparable to heart disease and cancer. As an example, it was estimated that in the United States the total monetary cost related to dementia in 2010 was approximately $200 billion [6]. 

Increasing scientific evidence indicates that AF and cognitive decline/dementia are frequently associated. These two conditions share many risk factors, such as hypertension, heart failure, diabetes and age. However, a causal relationship between AF (cause) and dementia (effect) has also been claimed [7]. In addition to the potential contribution of AF-related clinical cerebrovascular accidents (CVAs), there are several additional mechanistic hypotheses sustaining a causal association, which holds true also in the absence of CVAs: hemodynamic mechanisms (e.g., reduced mean cerebral blood flow, beat-to-beat alterations resulting in distal hypoperfusion and/or hypertensive events), subclinical microembolic cerebral infarctions, subclinical cerebral microbleeds (favored by oral anticoagulation treatment), and impaired cerebrovascular reactivity [8,9,10,11,12,13,14]. 

The present study provides an overview of AF and dementia burden, reporting the most recent data regarding incidence, prevalence, mortality, and morbidity of these two associated conditions, intended to grow in the upcoming decades. In addition, based on the epidemiological estimates derived from the Global Burden of Disease (GBD) study 2019, a potential correlation of the epidemiological trends of the two examined diseases was investigated.

## 2. Materials and Methods

GBD is a comprehensive regional and global research program of disease burden that assesses mortality and disability from major diseases, led by the Institute for Health Metrics and Evaluation (IHME) and collecting data from 1990 to present. In the GBD 2019, 282 causes of death, 369 diseases and injuries, and 87 risk factors for 204 countries and territories were included. A detailed description of the GBD study encompassing the included measures and the employed methodology may be found in the original publication [15]. Briefly, point estimates of incidence, prevalence, excess mortality and remission, along with uncertainty intervals, were assessed in DisModMR 2.1, a Bayesian meta-regression tool.

For the present study, data were downloaded from the open access GBD Results Tool (http://ghdx.healthdata.org/gbd-results-tool, accessed on 12 November 2021). Graphs and pictures were created with the GBD VizHub Tool (https://vizhub.healthdata.org/gbd-compare/, accessed on 12 November 2021)) and redistributed via a Creative Commons Attribution-NonCommercial-NoDerivatives 4.0 International License (https://creativecommons.org/licenses/by-nc-nd/4.0/, accessed on 12 November 2021). We evaluated worldwide estimates for “atrial fibrillation and flutter” (hereinafter referred to as atrial fibrillation—AF) and “Alzheimer’s disease and other dementias” (hereinafter referred to as dementia). International Classification of Diseases (ICD) codes are available online at http://ghdx.healthdata.org/record/ihme-data/gbd-2019-cause-icd-code-mappings, (accessed on 12 November 2021). The following metrics were evaluated:

- Incidence rate: number of new cases diagnosed (per 100,000 people);
- Prevalence rate: number of patients affected by the disease (per 100,000 people);
- Mortality rate: number of deaths due to the specific disease (per 100,000 people);
- Disability-adjusted life years (DALYs): sum of years lost due to premature death and years lived with disability (per 100,000 people)
- “All-ages” (crude) and “age-standardized” estimates were reported for incidence and prevalence data. Concerning mortality rate, the “all-ages” (crude) estimate was reported, along with the percentage impact of the cause-specific mortality rate on the overall mortality rate in the population. Finally, regarding DALYs data, we reported the “all-ages” (crude) estimate, as well as the percentage of the cause-specific DALYs on the overall DALYs in the worldwide population. We only reported point estimates, whereas uncertainty interval can be found at GBD Results tool (http://ghdx.healthdata.org/gbd-results-tool, accessed on 12 November 2021). We also provide specific subgroup data pertaining to low and high socio-demographic index (SDI) countries, to assess potential difference in AF and dementia epidemiology according to different socio-demographic scenarios.

The epidemiological trends of the examined diseases (AF and dementia) were assessed by evaluating the historical data from 1990 to 2019. In order to investigate the possible relationship between AF and dementia, the two crude incidence rate time-series were analyzed using the exploratory Granger test. This test was originally introduced in the economic field in 1969 in order to determine whether one time series is useful to forecast another [16]. It can be said that a variable *x* (in our case AF incidence) that evolves over time Granger-causes another evolving variable *y* (in our case dementia incidence) if predictions of the value of *y* based on its own past values and on the past values of *x* are better than predictions of Y based only on *y*’s own past values. The analyses were performed using R software version 4.0.3 (R Foundation for Statistical Computing, Vienna, Austria). The *lmtest* package version 0.9–39 was used to perform Granger tests [17]. A *p*-value of less than 0.05 was considered statistically significant.

## 3. Results

**Figure 1:**
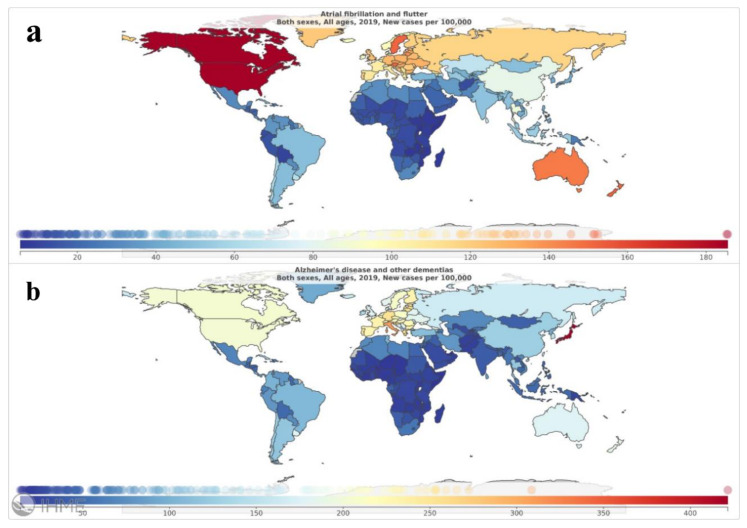
Global maps depicting crude incidence rate of the investigated conditions in 2019: panel (**a**), atrial fibrillation and atrial flutter; panel (**b**), Alzheimer’s disease and other forms of dementia. Source: https://vizhub.healthdata.org/epi/, (accessed on 12 November 2021).

**Figure 2:**
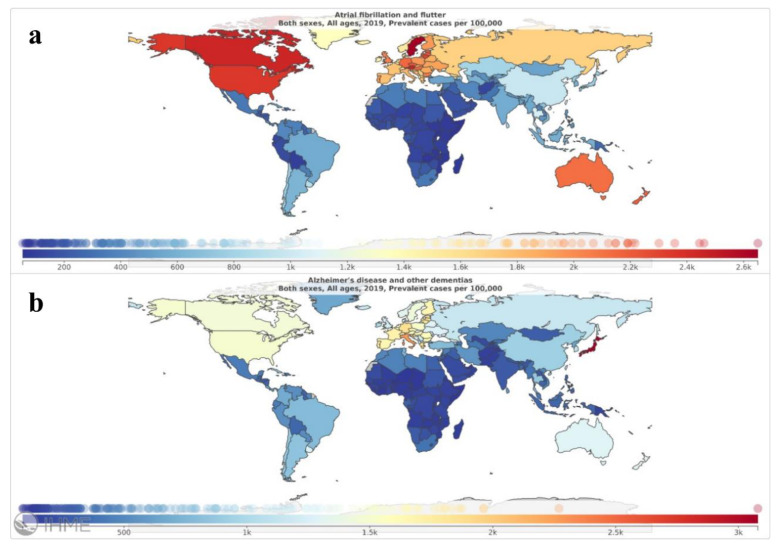
Global maps depicting crude prevalence rate of the investigated conditions in 2019: panel (**a**), atrial fibrillation and atrial flutter; panel (**b**), Alzheimer’s disease and other forms of dementia. Source: https://vizhub.healthdata.org/epi/, (accessed on 12 November 2021).

Detailed data regarding the analyzed metrics (incidence, prevalence, mortality rate and DALYs) for AF and dementia are reported in Table 1. Global maps reporting crude incidence and prevalence of the two conditions are reported in Figure 1 and Figure 2. Table 2 reports the subgroup data referred to low and high SDI countries. 

In 2019, AF was characterized by a incidence rate of 61.01 per 100,000 individuals (age-standardized estimate: 57.09 per 100,000 individuals), while prevalence rate settled at 771.51 per 100,000 individuals (age-standardized estimate: 743.47 per 100,000 individuals); AF-specific mortality rate was 4.08 per 100,000 individuals, accounting for 0.56% of the total mortality burden worldwide, while the rate of DALYs lost for AF amounted to 108.48 years per 100,000 individuals (0.33% of the total DALYs burden). Dementia was characterized by a slightly higher incidence rate (93.52 per 100,000 individuals; age-standardized estimate: 94.99 per 100,000 individuals) and a similar prevalence rate (667.20 per 100,000 individuals; age-standardized estimate: 682.48 per 100,000 individuals) compared to AF, while morbidity metrics were evidently higher for dementia (mortality rate: 20.89 per 100,000 individuals, 2.87% of the total mortality burden; rate of DALYs lost due to dementia: 326.68 years per 100,000 individuals, 1% of the total DALYs burden).

Comparing data from low and high SDI countries, evident differences are appreciated. In fact, crude estimates of all of the investigated metrics are higher in high SDI countries both for AF and dementia. For example, crude AF incidence rate is nearly seven times higher in high SDI countries compared to low SDI countries, while dementia is characterized by a ninefold increase in crude incidence rate. Similarly, the crude mortality and morbidity burden (mortality rate and rate of DALYs lost) of both conditions is 8–10 times higher in high SDI countries. Looking at age-standardized metrics we found that (albeit by a reduced magnitude) the estimates are still higher in high SDI countries if compared to lower ones. 

**Figure 3:**
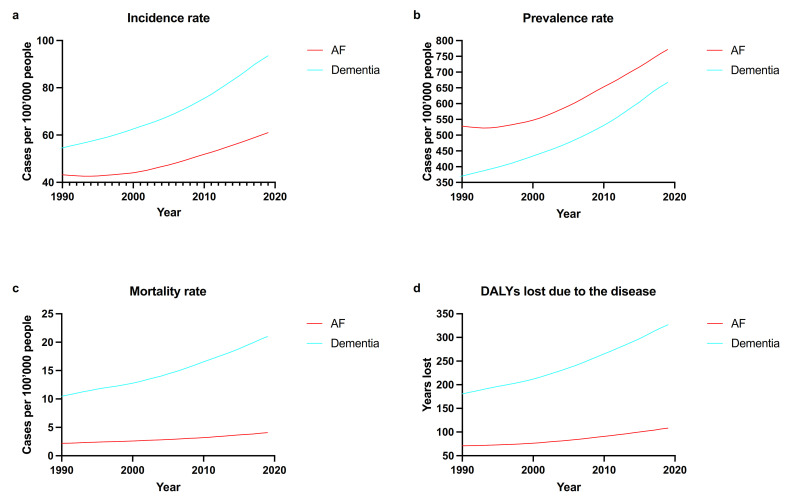
Global historical trends (1990–2019) for the crude estimates of the evaluated epidemiological metrics: panel (**a**), incidence rate; panel (**b**), prevalence rate; panel (**c**), mortality rate; panel (**d**), DALYs lost due to the disease.

Figure 3 reports the global historical trends (1990–2019) of the different crude metrics, for both the pathologies (age-standardized metrics can be found in Supplementary Figure S1). AF and dementia crude incidence rates progressively increased (from 43.24 to 61.01 per 100,000 individuals and from 54.60 to 93.52 per 100,000 individuals, respectively), with stable age-standardized estimates (from 58.54 to 57.09 per 100,000 individuals and from 93.58 to 94.99 per 100,000 individuals, for AF and dementia, respectively). The crude prevalence rate of AF increased (from 528.72 to 771.51 per 100,000 individuals), whereas the age-standardized prevalence rate did not show relevant changes (from 775.86 to 743.47 per 100,000 individuals). In the same timeframe, Alzheimer and other dementias have similarly experienced an increase in their crude prevalence rate (from 369.88 to 667.2 per 100,000 individuals), with a substantial stability of the age-standardized prevalence rate (from 645.89 to 682.48 per 100,000 individuals). Concerning mortality, a similar trend can be identified: AF-specific crude mortality rate increased (from 2.19 to 4.08 per 100,000 individuals), whereas the age-standardized rate was steady (from 4.29 to 4.38 per 100,000 individuals); dementia-specific crude mortality rate doubled (from 10.49 to 20.98 per 100,000 individuals), again with a substantial stability of the age-standardized estimates along the 29-year period (from 22.24 to 22.92 per 100,000 individuals).

**Figure 4:**
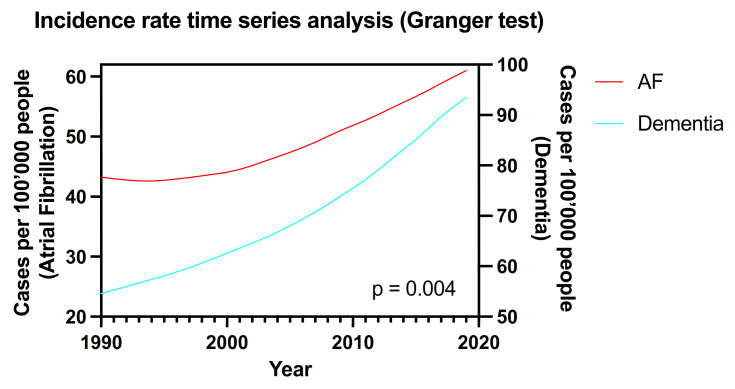
Granger test analysis on the global historical trends (1990–2019) of crude incidence rate of AF and dementia.

Finally, according to Granger test analysis performed on the crude incidence rate time-series of AF and dementia, we found a significant association between the two time trends (*p* = 0.004) as reported in Figure 4.

## 4. Discussion

The present study reports atrial fibrillation and dementia epidemiological data derived from the GBD Study 2019 and investigates the historical trend of these conditions (from 1990 to 2019). The main findings are:

In the last 30 years, the crude incidence and prevalence rate of AF and dementia increased by 41% and 71%, respectively. The consequent mortality and morbidity burden also doubled in the same timeframe, highlighting a progressively greater burden of these diseases.

Age-standardized metrics, which take into account the progressive aging of the global population in the previous decades, show substantial stability in terms of incidence and prevalence of both conditions, as well as in mortality and morbidity; if, on the one hand, these data suggest that the inflation of the crude estimates are driven mainly by population aging, on the other hand they highlight that further efforts are needed to prevent these conditions through stricter risk factor control.

Stratification by SDI suggests that these two conditions are clearly more frequently encountered in high SDI countries, not only due to longer life expectancy but also due to higher risk factors burden (as suggested by the fact that also age-standardized estimates, which intrinsically eliminate the confounding effect of the age-population structure, are greater for high compared to lower SDI countries). In particular, AF and dementia represent a higher relative share on total mortality and morbidity in high SDI countries.

Finally, the explorative analysis using the Granger test on the historical trends of the incidence rate of the two conditions confirms a correlation between the two diseases. Although this analysis cannot prove a causal relationship, it suggests that a close relationship between these two conditions exists, highlighting that any future increase in AF incidence would easily be followed by a similar increase in dementia. 

A cross-sectional analysis of a subgroup of patients from the Rotterdam Study, published more than 20 years ago, showed for the first time an independent association between AF and dementia. In this seminal study, the risk of dementia/cognitive impairment was nearly twice in AF patients compared to non-AF individuals [18]. 

In fact, the potential correlation of the two diseases can be explained by several mechanisms. Since AF is one of the main causes of ischemic stroke, a vascular type of cognitive impairment/dementia may surely candidate for a central role in this association. 

Similarly, advanced interatrial block (A-IAB), defined at ECG as *p*-wave duration ≥120 ms with biphasic morphology in inferior leads, is a marker of severe atrial disease. Indeed, A-IAB, as well as AF, is a manifestation of atrial cardiomyopathy [19,20], and stroke and microembolic events may be, at least partially, caused by the atrial disease and not only by the rhythm alteration. In fact, A-IAB has been found to be independently associated with onset of AF, stroke and, more specifically, cognitive decline [21,22,23]. Based on these data, further studies are necessary to evaluate whether the presence of A-IAB confers *per se* an increased risk of stroke or whether this association is mediated by the occurring of subclinical AF episodes. In the former case, A-IAB and AF could be considered as clinically equivalent, and randomized trials evaluating the role of oral anticoagulation for the prevention of stroke and dementia, whose indication should be driven by clinical factors, such as CHA2DS2-VASc score, and, possibly, by imaging data (e.g., left atrial fibrosis at MRI or left atrial strain at echocardiography) would be necessary. As a matter of fact, AF patients present a 30% increased risk of developing dementia regardless of clinical cerebrovascular events (transient ischemic attacks or strokes) [24]. Moreover, AF and dementia share several risk factors, including hypertension, diabetes, inflammatory diseases, altered lipid profile, physical inactivity, and ageing [25,26,27].

Therefore, other mechanisms can potentially further explain this relationship. The inflammatory states associated with AF have been advocated in the genesis of cognitive decline; inflammatory markers elevation is typical in AF patients, and relates to a prothrombotic state [28]. Moreover, inflammatory cytokines, released after cerebral vascular damage, can increase the production of amyloid precursor protein, whose misfolded oligomeric forms are toxic to the brain cells and are implicated in Alzheimer’s disease [29,30,31]. In addition, AF can lead to silent cerebral ischemias (SCI), which are twice as common in AF compared to sinus rhythm patients [12]. These lesions can be found at magnetic resonance imaging (MRI) and are directly associated with cognitive impairment [11,12]. AF can also cause microbleeds, possibly facilitated by anticoagulation treatment, whose presence and number are correlated to a reduced cognitive function [32,33]. Finally, the hemodynamic effects of AF on systemic and cerebral flow might play an active role; the loss of the atrial systole is responsible for a 20–30% reduction in cardiac output [34], particularly impacting in elderly subjects with impaired cerebral blood flow autoregulation [35], and AF-related cerebrovascular dysfunction [13]. Indeed, patients with AF present lower mean cerebral blood flow than in age-matched controls, particularly in cases of persistent AF [36,37]. In addition, a computational model simulating AF R-R interval variability has documented beat-to-beat effects resulting in transient cerebral hypoperfusion and hypertensive events in the deep cerebral circle [8,9]. The latter hypothesis was also recently validated in vivo using spatially resolved cerebral near infrared spectroscopy (SRS-NIRS). Elective electrical cardioversion restoration sinus rhythm in persistent AF patients, significantly reduced beat-to-beat hypoperfusion and hypertensive events in the cerebral microcirculation, assessed by inter-beat differences of tissue hemoglobin index, an indirect index of tissue perfusion [10]. 

Definitive evidence regarding the optimal strategy to prevent dementia in patients with AF is lacking. However, increasing scientific evidence suggests that a rhythm control strategy aiming at sinus rhythm maintenance, on top of a proper oral anticoagulation, may prevent (or at least delay) cognitive decline/dementia occurrence. Waiting for randomized clinical trials to shed light on this topic, several observational data are presently available, largely focusing on the role of catheter ablation (the most effective rhythm control approach) in preventing cognitive decline [38]. A retrospective analysis conducted on 194,928 patients registered in the Korean National Health Insurance Service database reported that, over a median follow-up of more than four years, catheter ablation was associated with lower rate of overall dementia compared to patients medically treated after censored for stroke (HR 0.76). This reduction was evident in case of ablation success, while ablation failure, with recurrence of the arrhythmia, was not associated with significant differences [39]. Another retrospective study reported, over nine years follow up, a lower incidence of new onset dementia in patients who had undergone catheter ablation compared to patients with AF who were not recommended for catheter ablation (HR 0.44), especially in those aged >65 years old (HR 0.46) [40]. The Montreal cognitive assessment score also reported improved cognitive performance within an AF catheter ablation group compared to patients treated medically. Interestingly, cognitive improvement was more evident in patients who presented pre-ablation cognitive impairment (OR 13.70, 95%CI 4.83–38.87) [41]. Eventually AF catheter ablation has shown to reduce the risk of stroke/TIA and dementia (HR 0.51, *p* = 0.04) [42], and to confer to the patient a post-ablation dementia risk similar to that of the general population free from AF [43]. All of this evidence is pointing toward a potential reduction of AF-related dementia incidence when sinus rhythm maintenance is pursued with effective treatment, lay the ground for dedicated, properly powered, randomized studies assessing whether catheter ablation-based rhythm control strategy might help in breaking the causal chain between AF and cognitive decline/dementia.

## 5. Conclusions

Atrial fibrillation and dementia have seen an important rise in their incidence and prevalence, as well as in their mortality and morbidity burden, in the last three decades, particularly due to population aging. Future epidemiological predictions foresee a further worsening of this epidemiological situation. Given the evidence suggesting a causative link between atrial fibrillation and subsequent cognitive decline/dementia, it is imperative, in the near future, to assess the ability of different AF management strategies in order to break the vicious causal chain and limit the share of AF-related dementia.

## Figures and Tables

**Table 1:** Epidemiological metrics of atrial fibrillation and dementia in 2019 estimated by the GBD Study 2019.

Measure	Crude Estimate (Per 100,000 Individuals)	Age-Standardized Estimate (Per 100,000 Individuals)	Percentage on Total Deaths or DALYs
**AF**			
Incidence rate	61.01	57.09	NA
Prevalence rate	771.51	743.47	NA
Mortality rate	4.08	4.38	0.56%
DALYs	108.48	107.13	0.33%
**Dementia**			
Incidence rate	93.52	94.99	NA
Prevalence rate	667.20	682.48	NA
Mortality rate	20.98	22.92	2.87%
DALYs	326.68	338.64	1.00%

AF, atrial fibrillation; DALYs, disability-adjusted life years; NA, not applicable.

**Table 2:** Focus on 2019 epidemiological metrics of atrial fibrillation and dementia in low and high SDI countries estimated by the GBD Study.

Measure	Crude Estimate (Per 100,000 Individuals)	Age-Standardized Estimate (Per 100,000 Individuals)	Percentage on Total Deaths or DALYs
**AF**			
Incidence rate			
Low SDI	18.37	41.97	NA
High SDI	121.89	69.22	NA
Prevalence rate			
Low SDI	199.58	514.20	NA
High SDI	1733.59	895.72	NA
Mortality rate			
Low SDI	1.17	4.30	0.17%
High SDI	10.85	4.61	1.24%
DALYs			
Low SDI	32.86	91.91	0.07%
High SDI	248.40	122.64	0.84%
Dementia			
Incidence rate			
Low SDI	24.23	76.97	NA
High SDI	219.65	100.57	NA
Prevalence rate			
Low SDI	161.94	514.75	NA
High SDI	1598.23	721.43	NA
Mortality rate			
Low SDI	5.50	23.03	0.80%
High SDI	55.58	22.66	6.3%
DALYs			
Low SDI	93.13	315.76	0.20%
High SDI	755.44	332.40	2.56%

AF, atrial fibrillation; DALYs, disability-adjusted life years; NA, not applicable; SDI, socio-demographic index.

## Data Availability

All data are freely available at http://ghdx.healthdata.org/gbd-results-tool, (accessed on 12 November 2021).

## Supplementary Materials

The following supporting information can be downloaded at: https://www.mdpi.com/article/10.3390/medicina58030361/s1. Figure S1: Global historical trends (1990–2019) for the age-standardized estimates of the evaluated epidemiological metrics: panel A, incidence rate; panel B, prevalence rate; panel C, mortality rate; panel D, DALYs lost due to the disease.
