# Supplementary material for: Atrial Fibrillation and Dementia: Epidemiological Insights on an Undervalued Association
Source: Medicina (Kaunas). 2022 Mar 1;58(3):361. doi: 10.3390/medicina58030361 (PMC8955523; doi:10.3390/medicina58030361)

# Atrial fibrillation and dementia: epidemiological insights on an undervalued association

## *Supplementary Material*

**Figure S1** Global historical trends (1990–2019) for the age-standardized estimates of the evaluated epidemiological metrics: panel (**a**), incidence rate; panel (**b**), prevalence rate; panel (**c**), mortality rate; panel (**d**), DALYs lost due to the disease.

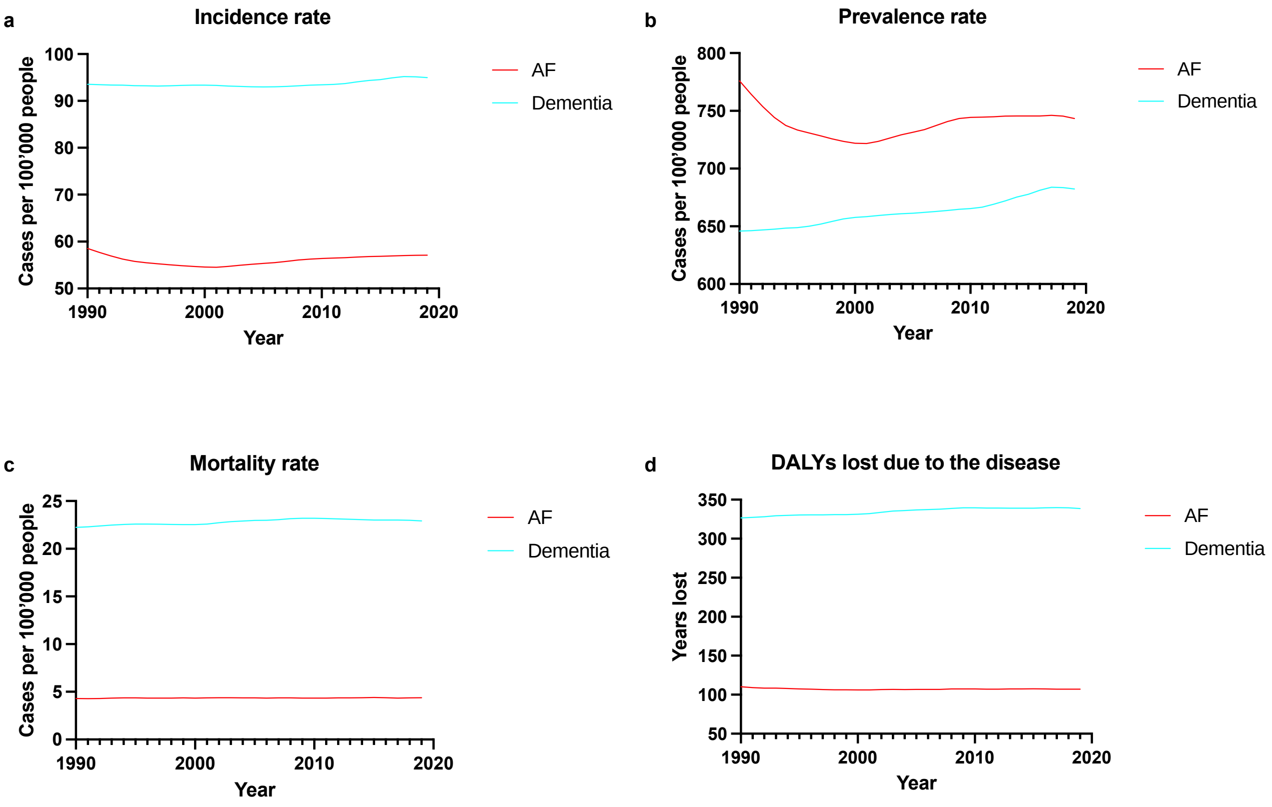

Supplement: Supplementary file 1 [file medicina-58-00361-s001.zip › medicina-1566897-supplementary.pdf]
